# Association between blood total mercury and psoriasis: The NHANES 2005–2006 and 2013–2014: A cross-sectional study

**DOI:** 10.1371/journal.pone.0309147

**Published:** 2024-10-15

**Authors:** Yanan Tuo, Yali Li, Tao Guo

**Affiliations:** Department of Dermatology, Tianjin Academy of Traditional Chinese Medicine Affiliated Hospital, Hongqiao District, Tianjin, China; City College of New York, UNITED STATES OF AMERICA

## Abstract

An inflammatory skin condition called psoriasis results from immune system interactions that are out of balance. Reactive oxygen species are produced as a general mechanism of mercury toxicity. This study aimed to determine whether there was an association between blood total mercury and psoriasis in US adults. Utilizing data from the National Health and Nutrition Examination Survey (NHANES) 2005–2006 and 2013–2014. NHANES is a national research survey program every two years to assess the population’s nutritional and physical health. The relationship between blood total mercury and psoriasis was studied using multivariable logistic regression models and smooth curve fitting. Subgroup analysis and interaction tests were used to investigate if this association was stable across populations. After adjusting for several factors, we found a positive association between blood total mercury and psoriasis in 6086 participants. According to the fully adjusted model, each 1-unit increase in blood total mercury was associated with an 8% increase in the prevalence of psoriasis [1.08 (1.03, 1.14)]. The favorable association seems to be more pronounced in non-diabetes. Our research shows a positive association between psoriasis and blood total mercury in US adults. The results of this study need to be supported by additional prospective research.

## Introduction

Psoriasis is a long-term inflammatory skin condition that manifests as distinct, scaly plaques. Approximately 125 million individuals worldwide have psoriasis [1]. The patient’s quality of life (QoL) is significantly impacted by psoriasis, a multisystemic condition [2, 3]. Patients with psoriasis have been found to have higher rates of cardiovascular morbidity and mortality due to systemic chronic inflammation [4, 5]. Additionally, concurrent conditions such as psoriatic arthritis, metabolic syndrome, diabetes, cardiovascular disease, nephropathy, gastrointestinal disorders, and brain disorders may be linked to psoriasis [6–10]. Furthermore, psoriasis has been linked to several different inflammasomes, inflammasome-related genes, and genetic susceptibility loci [11].

Mercury is a highly reactive toxic heavy metal with no known physiological effects. Mercury exposure may predispose people to many diseases by promoting the production of free radicals [12]. According to the reports, mercury causes an increase in tumor necrosis factor and interleukin, which in turn causes free radical generation, oxidative stress, thrombosis, and vascular inflammation [13]. Mercury can increase the risks of diabetes and metabolic syndrome [14]. However, it’s still unclear among American communities if mercury exposure has an association with psoriasis.

Even in relatively small amounts, the poisonous heavy metal mercury accumulation can negatively affect human health [15]. Due to several mechanisms of action, including the potential modification of gene expression, exposure to certain metals may negatively affect human health. The control of intracellular redox homeostasis is demonstrated to be impaired by mercury, leading to an increase in intracellular oxidative stress [16]. Reduced glutathione peroxidase (GPx) activity and glutathione (GSH) depletion may be the causes of the mercury-induced increase in H2O2 levels [17]. Heavy metals and skin diseases, including atopic dermatitis and acne vulgaris, have been linked by certain writers [18, 19]. Numerous recent studies have demonstrated the association between environmental factors and skin problems. Blood mercury is a frequently used biomarker of mercury exposure [20, 21], but the association between blood total mercury and psoriasis has not been investigated. So, we carried out a cross-sectional study to explore the relationship between blood total mercury and psoriasis utilizing information from the 2005–2006 and 2013–2014 National Health and Nutrition Examination Survey (NHANES).

## Materials and methods

### Study population

**Fig 1:**
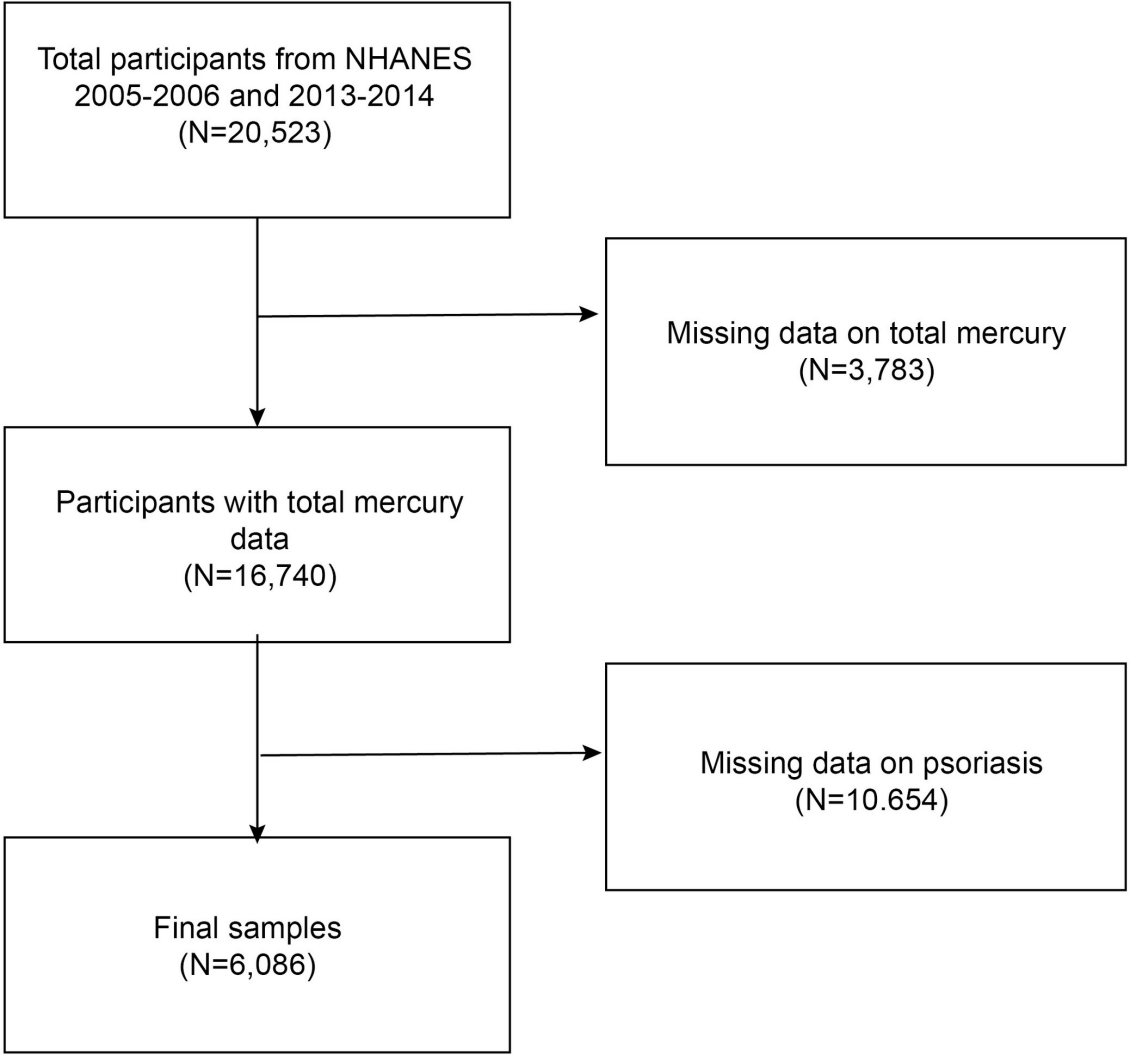
The selection flow chart for participants. NHANES, National Health and Nutrition Examination Survey.

The NHANES is a nationally representative survey by the Centers for Disease Control and Prevention [22, 23]. The study’s methodology was approved by the National Center for Health Statistics (NCHS) Research Ethics Review Board. All participants gave their written consent when recruiting [24, 25]. For our cross-sectional analysis, we used NHANES data from the 2005–2006 and 2013–2014 cycles, corresponding to the years when data on psoriasis were gathered. In response to the inquiry, "Have you ever been told by a health care provider that you had psoriasis?" psoriasis was self-reported. Our study included 20,523 people. We disqualified 3,783 people with missing blood total mercury data and 10,654 people with missing psoriasis data from the 20,523 eligible adults. In the end, 6,086 participants participated in the survey (Fig 1).

### Data on biomarkers

The National Center for Environmental Health, Division of Laboratory Sciences, Centers for Disease Control and Prevention, Atlanta, GA, receives, processes, stores, and ships serum specimens for examination. Inductively coupled plasma dynamic reaction cell mass spectrometry determines the total blood mercury level. The 1988 Clinical Laboratory Improvement Act requirements are satisfied by the NHANES quality assurance and control procedures.

### Diagnosis of psoriasis

In response to the inquiry, "Have you ever been told by a health care provider that you had psoriasis?" psoriasis was self-reported.

### Covariates

The relationships between blood total mercury and psoriasis may be obscured by covariates in multivariable models. Age, gender, race, alcohol drinking status, smoking status, body mass index, poverty to income ratio, education, diabetes status, waist circumference, total cholesterol, triglyceride, LDL- cholesterol, HDL- cholesterol, serum glucose, serum total bilirubin, white blood cell count, high blood pressure, total cholesterol, cadmium, lead were all variables in this research.

### Statistical analysis

Student’s t-tests were used to compare continuous variables with normal distribution between groups with psoriasis and those without. The continuous variables were defined as mean ± standard deviation. The χ2 test was used to compare the two groups for categorical variables expressed as percentages. Using univariate analysis, the risk of psoriasis was assessed for each predictive variable. The association between blood total mercury and psoriasis was examined using logistic regression analyses. After transforming blood total mercury from a continuous variable to a categorical variable (quartile) a trend test was used to investigate the trend of association between blood total mercury and psoriasis. Model 1 had no adjusted variables, Model 2 had adjusted age, gender, race, education, and annual family income, and Model 3 had adjusted every covariate in Table 1. Subgroup analyses in terms of age, gender, diabetes, and BMI were conducted to examine the presence of significant interactions of these covariates with the association between blood total mercury and psoriasis. Moreover, stratified factors were considered as possible effect modifiers. To assess the heterogeneity using the likelihood ratio test, an interaction term was used. The odds ratio (OR), 95% confidence interval (CI), and p-value are displayed along with the results. Smoothing curve fitting was used to explore the nonlinear association between blood total mercury and psoriasis. Statistics were regarded as significant when two-tailed p 0.05. There are no missing values for age, sex, race, high blood pressure, diabetes status, lead, and cadmium. Education, poverty to income ratio, white blood cell count, body mass index, waist circumference, serum glucose, serum total bilirubin, HDL- cholesterol, triglyceride, and total cholesterol, had missing values of 5.01%, 5.59%, 0.18%, 0.87%, 0.72%, 1.13%, 1.20%, 0.80%, 0.80% and 0.80%, respectively, which were interpolated using the mean value. Alcohol drinking and smoking status, had missing values of 9.99% and 2.51%, respectively, which were interpolated using the median value. For all statistical analyses, we utilized R (http://www.r-project.org) and EmpowerStats (http://www.empowerstats.com).

**Table 1:** Characteristics of participants in the NHANES 2005–2006 and 2013–2014 cycles.

	Psoriasis (N = 150)	Never diagnosed with psoriasis (N = 5936)	*P-*value
Age(years)	44.27 ± 15.12	41.61 ± 16.11	0.022
Serum glucose (mg/dL)	100.51 ± 37.42	99.17 ± 35.62	0.799
Serum total bilirubin (mg/dL)	0.68 ± 0.34	0.66 ± 0.34	0.763
White blood cell count (1000 cells/uL)	7.59 ± 2.19	7.45 ± 2.35	0.316
Total cholesterol (mg/dL)	198.05 ± 42.04	193.01 ± 42.80	0.205
Triglyceride (mg/dL)	163.94 ± 212.66	127.96 ± 99.58	0.020
LDL-cholesterol (mg/dL)	119.26 ± 40.85	112.33 ± 35.99	0.188
Direct HDL-Cholesterol (mg/dL)	53.77 ± 17.71	54.01 ± 16.53	0.433
Body Mass Index (kg/m**2)	29.91 ± 7.05	28.82 ± 7.18	0.028
Waist Circumference (cm)	100.55 ± 17.31	97.41 ± 16.47	0.025
Cadmium (ug/L)	0.55 ± 0.75	0.48 ± 0.57	0.105
Lead (ug/dL)	1.35 ± 1.09	1.44 ± 1.38	0.759
Mercury, total (ug/L)	1.92 ± 3.46	1.50 ± 2.28	0.027
Gender, % (N)			0.168
Male	41.33(62)	47.02(2,791)	
Female	58.67(88)	52.98(3,145)	
Race, % (N)			<0.001
Mexican American	5.33(8)	19.26(1,143)	
Other Hispanic	4.67(7)	6.45(383)	
Non-Hispanic White	61.33(92)	43.55(2,585)	
Non-Hispanic Black	16.00(24)	21.34(1,267)	
Other Race-Including Multi-Racial	12.67(19)	9.40(558)	
Education, % (N)			0.249
Less Than 9th Grade	2.72(4)	9.05(510)	
9-11th Grade(Includes 12th grade with no diploma)	14.29(21)	13.74(774)	
High School Grad/GED or Equivalent	24.49(36)	22.61(1,274)	
Some College or AA degree	31.29(46)	31.47(1,773)	
College Graduate or above	27.21(40)	23.13(1,303)	
Family PIR	2.82 ± 1.71	2.61 ± 1.65	0.125
Smoke at least 100 cigarettes in life, % (N)			0.003
Yes	56.38(85)	42.32(2,448)	
No	43.62(65)	57.68(3,336)	
Diabetes, % (N)			<0.0001
Yes	10.00(15)	8.09(480)	
No	82.67(124)	89.76(5,328)	
Borderline	6.67(10)	2.06(122)	
Don’t know	0.67(1)	0.10(6)	
Had at least 12 alcoholic drinks/1 year, % (N)			0.016
Yes	77.21(105)	70.50(3,766)	
No	22.06(30)	29.41(1,571)	
Don’t know	0.74(1)	0.09(5)	
High blood pressure, % (N)			0.014
Yes	35.33(53)	26.67(1,583)	
No	64.67(97)	73.33(4,353)	

Mean ± SD for continuous variables, the P value was produced by the weighted linear regression model; (%) with relation to categorical variables: the P value was determined by the weighted chi-square test. There are no missing values for age, sex, race, high blood pressure, diabetes status, lead, and cadmium. Education, poverty to income ratio, white blood cell count, body mass index, waist circumference, serum glucose, serum total bilirubin, HDL- cholesterol, triglyceride, and total cholesterol, had missing values of 5.01%, 5.59%, 0.18%, 0.87%, 0.72%, 1.13%, 1.20%, 0.80%, 0.80% and 0.80%, respectively, which were interpolated using the mean value. Alcohol drinking and smoking status, had missing values of 9.99% and 2.51%, respectively, which were interpolated using the median value. Abbreviation: LDL, low-density lipoprotein; HDL, high-density lipoprotein; PIR, Poverty income ratio.

### Ethical approval

The NCHS Ethics Review Board authorized all aspects of this study that involved using human subjects, human materials, or human data in conformity with the Declaration of Helsinki.

## Results

### Baseline characteristics

Of the 20,523 participants, 14,437 were disqualified because their blood total mercury and psoriasis data were either absent or improbable, leaving 6,086 participants for analysis. A total of 150 participants (2.46%) had psoriasis, and 5,936 (97.54%) did not have psoriasis. Of 6,086 participants, the mean (SD) age was 41.67 (12.14) years, with 53.12% female and 43.97% non-Hispanic white. Participants with psoriasis were older (mean age, 44.27years) than those without psoriasis (mean age, 41.61 years), were more likely to be non-Hispanic White individuals (61.33% vs. 43.55%), and were more probable to have a higher level of triglyceride, BMI, waist circumference and blood total mercury. In addition, participants with psoriasis were more likely to have a higher likelihood of diabetes, high blood pressure, smokers, and drinkers. There were no significant differences between the two groups in gender, education, annual family income, serum glucose, serum total bilirubin, white blood cell count, total cholesterol, LDL-cholesterol, direct HDL-Cholesterol, cadmium and lead (Table 1).

### Univariate analysis for psoriasis

The relationship between univariate variables and psoriasis is shown in Table 2. In the univariate analysis, age (OR: 1.01, 95% CI: 1.00–1.02, p = 0.0462), triglyceride (OR: 1.00, 95% CI: 1.00–1.00, p = 0.0059), Body Mass Index (OR: 1.02, 95% CI:1.00–1.04, p = 0.0271), waist Circumference (OR: 1.01, 95% CI: 1.00–1.02, p = 0.0249), total mercury (OR: 1.05, 95% CI: 1.01–1.10, p = 0.0297) were associated with an increased risk of psoriasis. Moreover, those with psoriasis also had increased risk factors for diabetes, hypertension, smoking, and alcohol consumption. However, there was no indication that Serum glucose, serum total bilirubin, white blood cell count, total cholesterol, LDL-cholesterol, Direct HDL-cholesterol, cadmium, lead, gender, or family PIR was significantly associated with psoriasis risk.

**Table 2:** Univariate analysis for psoriasis.

Covariate	Statistics	OR (95% CI)	*P-*value
Age(years)	41.67 ± 16.10	1.01 (1.00, 1.02)	0.0462
Serum glucose (mg/dL)	99.20 ± 35.67	1.00 (1.00, 1.01)	0.6490
Serum total bilirubin (mg/dL)	0.67 ± 0.34	1.11 (0.75, 1.63)	0.6044
White blood cell count (1000 cells/uL)	7.45 ± 2.35	1.02 (0.96, 1.09)	0.4548
Total cholesterol (mg/dL)	193.13 ± 42.79	1.00 (1.00, 1.01)	0.1532
Triglyceride (mg/dL)	128.84 ± 103.87	1.00 (1.00, 1.00)	0.0059
LDL-cholesterol (mg/dL)	112.50 ± 36.12	1.01 (0.98, 1.01)	0.1171
Direct HDL-Cholesterol (mg/dL)	54.00 ± 16.56	1.00 (0.99, 1.01)	0.8617
Body Mass Index (kg/m**2)	28.84 ± 7.18	1.02 (1.00, 1.04)	0.0271
Waist Circumference (cm)	97.48 ± 16.49	1.01 (1.00, 1.02)	0.0249
Cadmium (ug/L)	0.48 ± 0.57	1.19 (0.94, 1.50)	0.1429
Lead (ug/dL)	1.43 ± 1.37	0.95 (0.82, 1.09)	0.4322
Mercury, total (ug/L)	1.51 ± 2.31	1.05 (1.01, 1.10)	0.0297
Gender			0.1691
Male(%)	46.88	Reference	
Female(%)	53.12	1.26 (0.91, 1.75)	
Race			<0.001
Mexican American	18.91	Reference	
Other Hispanic	6.41	2.61 (0.94, 7.25)	
Non-Hispanic White	43.99	5.08 (2.46, 10.51)	
Non-Hispanic Black	21.21	2.71 (1.21, 6.05)	
Other Race-Including Multi-Racial	9.48	4.86 (2.12, 11.18)	
Education			0.0223
Less Than 9th Grade(%)	8.89	Reference	
9-11th Grade(Includes 12th grade with no diploma)(%)	13.75	3.46 (1.18, 10.14)	
High School Grad/GED or Equivalent(%)	22.66	3.60 (1.28, 10.17)	
Some College or AA degree(%)	31.47	3.31 (1.19, 9.23)	
College Graduate or above(%)	23.23	3.92 (1.40, 11.02)	
Family PIR	2.61 ± 1.65	1.08 (0.98, 1.19)	0.1253
Smoke at least 100 cigarettes in life			0.0007
Yes(%)	42.68	Reference	
No(%)	57.32	0.57 (0.41, 0.79)	
Diabetes			0.0219
Yes(%)	8.13	Reference	
No(%)	91.87	0.74 (0.43, 1.28)	
Had at least 12 alcoholic drinks/1 year			0.0204
Yes(%)	70.66	Reference	
No(%)	29.23	0.68 (0.45, 1.03)	
Don’t know(%)	0.11	7.17 (0.83, 61.94)	
High blood pressure			0.0167
Yes(%)	26.88	Reference	
No(%)	73.12	0.66 (0.47, 0.93)	

Abbreviation: LDL, low-density lipoprotein; HDL, high-density lipoprotein; PIR, Poverty income ratio.

### Association between blood total mercury and psoriasis

**Fig 2:**
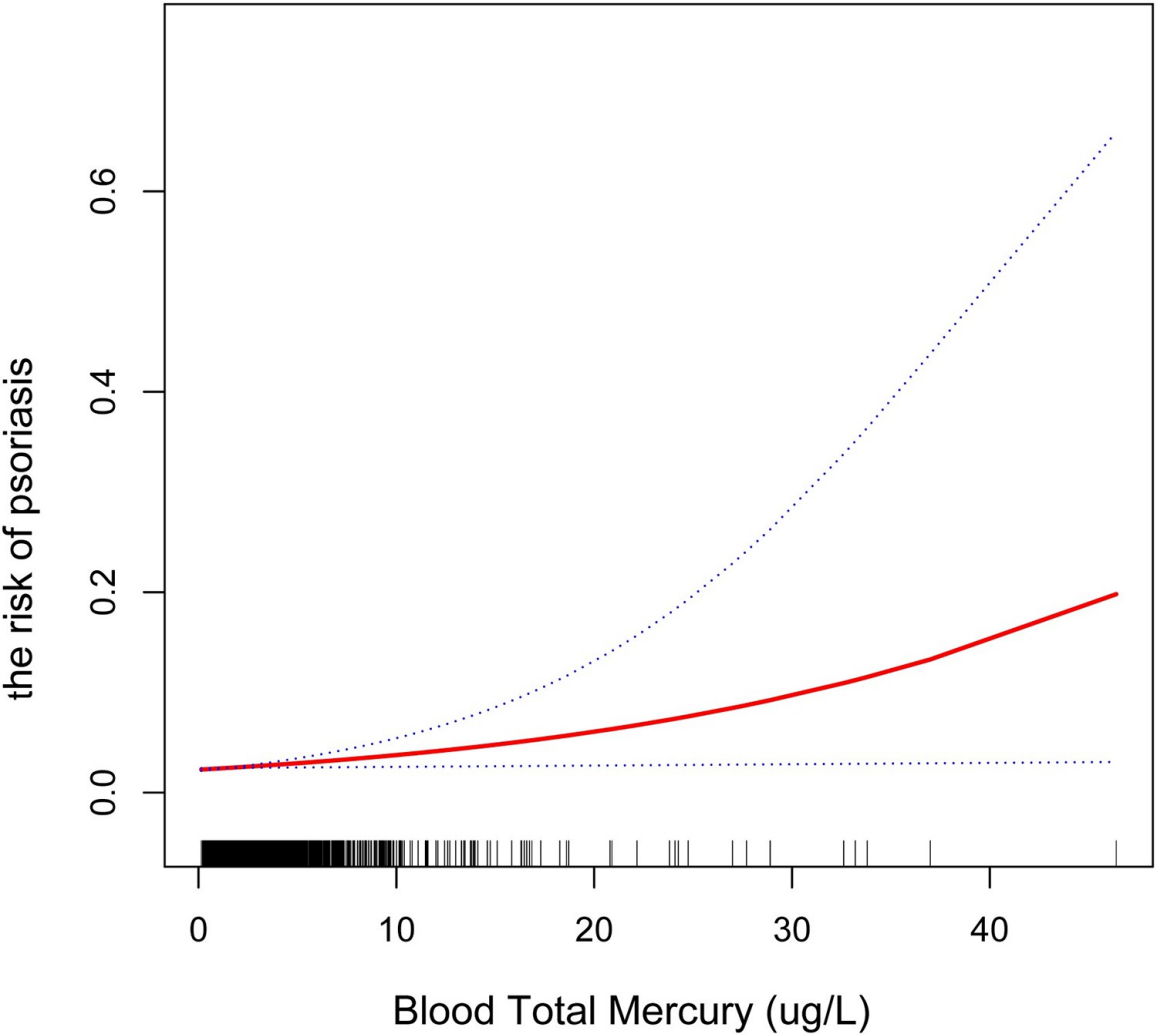
The exposure-response analyses between total blood mercury and psoriasis. The 95% confidence interval and the estimated odds ratio are shown by the upper, lower, and center lines, respectively.

Table 3 displays the correlations between blood total mercury and psoriasis. The unadjusted model positively linked Blood total mercury with psoriasis [1.05 (1.01, 1.10)]. And this correlation is also significant after adjusting for partial covariates in Model 2 [1.05(1.00, 1.10)], as well as in the fully adjusted Model 3 [1.08 (1.03, 1.14)]. After adjusting for all covariates, the risk of developing psoriasis increased by 8% for each 1-unit increase in blood total mercury. The blood total mercury was categorized into quartiles, and this did not affect the statistical significance of the above association (all P for trend < 0.05). Participants in the highest quartile of blood total mercury had a 45% increased risk of developing psoriasis [1.45 (1.10, 2.38)] compared to participants in the lowest. The outcomes of the smooth curve fitting also confirmed the positive connection between blood total mercury and psoriasis (Fig 2).

**Table 3:** Associations between blood total mercury and psoriasis.

blood total mercury	Psoriasis	P-value
	OR (95% CI)	
Crude model (Model 1) Continuous	1.05 (1.01, 1.10)	0.0297
Categories Quartile 1 Quartile 2 Quartile 3 Quartile 4 P for trend Minimally adjusted model (Model 2) Continuous Categories Quartile 1 Quartile 2 Quartile 3 Quartile 4 P for trend Fully adjusted model (Model 3) Continuous Categories Quartile 1 Quartile 2 Quartile 3 Quartile 4	1 (ref) 1.15 (0.71, 1.85) 1.18 (0.74, 1.90) 1.38 (1.11, 2.18) 0.018 1.05(1.00, 1.10) 1 (ref) 1.15 (0.71, 1.85) 1.18 (0.73, 1.90) 1.39 (0.87, 2.20) 0.017 1.08 (1.03, 1.14) 1 (ref) 1.01 (0.60, 1.71) 1.07 (0.64, 1.81) 1.45 (1.10, 2.38)	0.5797 0.4875 0.0173 0.0253 0.5815 0.4974 0.1644 0.0035 0.9692 0.7891 0.0145
P for trend	0.018	

Model 1: no covariates were adjusted. Model 2: age, gender, race were adjusted. Model 3: age, gender, race, serum glucose, serum total bilirubin, White blood cell count, total cholesterol, triglyceride, LDL-cholesterol, HDL-cholesterol, Body Mass Index, waist Circumference, cadmium, lead, education, annual family income, drinking alcohol, diabetes, high blood pressure, smoking were adjusted.

### Subgroup analyses

We performed subgroup analysis and interaction tests stratified by gender, age, BMI, and diabetes to determine whether the association between blood total mercury and psoriasis was constant across the general population and to spot any potential disparities in population settings (Table 4). Our results showed that the association between blood total mercury and psoriasis significantly differed between diabetes subgroups (P for interaction < 0.05). The risk of developing psoriasis in non-diabetic adults increased by 5% per 1 unit increase in blood total mercury. In contrast, among those adults with diabetes, the association between blood total mercury and psoriasis became a non-significant negative association.

**Table 4:** Subgroup analysis of the association between blood total mercury and psoriasis.

Subgroup	Psoriasis [OR (95%CI)]	*P* for interaction
Sex		0.7488
Male	1.06 (0.96, 1.17)	
Female	1.09 (0.94, 1.26)	
Age		0.1318
< 60 years	1.12 (1.02, 1.23)	
≥ 60 years	0.83 (0.47, 1.47)	
Diabetes, (%)		0.0386
Yes	0.84(0.55, 1.28)	
NO	1.05 (1.01, 1.11)	
BMI		0.8515
<24.9 kg/m^2^	1.06 (0.89, 1.27)	
25–29.9 kg/m^2^	1.06 (0.90, 1.25)	
≥30 kg/m^2^	1.12 (0.97, 1.28)	

age, gender, race, serum glucose, serum total bilirubin, White blood cell count, total cholesterol, triglyceride, LDL-cholesterol, HDL-cholesterol, Body Mass Index, waist Circumference, cadmium, lead, education, annual family income, drinking alcohol, diabetes, high blood pressure, smoking were adjusted.

## Discussion

In the cross-sectional survey with 6,086 representative participants, we discovered the positive associations between blood total mercury and psoriasis, and there was a significant dependence of diabetes on this association, indicating that higher levels of blood total mercury may lead to an increased risk of developing psoriasis, especially in the non-diabetic population. Our findings suggest that blood total mercury may have potential clinical value in diagnosing psoriasis risk and disease severity.

As far as we know, only some research has examined the relationship between mercury exposure and psoriasis development. Mercury levels were more significant in the blood of vitiligo (1.45 1.26 g/L) and psoriasis (1.78 1.24 ng/ml) patients compared to the control group, according to a recent study, but the difference was not significant [16]. While the results of this study did not support a link between mercury exposure and psoriasis, this research cannot exclude the possibility that higher exposures than those observed in our sample may be positively associated with psoriasis. There is likewise little evidence linking mercury exposure to psoriasis in other areas. Mercury is thought to be a significant contributing factor to autoimmune disorders, particularly the overproduction of specific antinuclear antibodies [26]. There have been some theories that suggest mercury exposure may raise the likelihood of developing obesity. An Iran cross-sectional study of 320 adolescents discovered that adolescents with metabolic syndrome had greater mean (SD) mercury contents than adolescents without it [27]. Additionally, research has linked the severity of psoriasis to obesity and has shown that having a higher BMI increases the likelihood of developing psoriasis [28]. The general public is mainly exposed to mercury through contaminated foods that accumulate mercury. Due to earlier research suggesting that mercury could emerge as a possible obesogenic property, there is one more evidence of why mercury and diets are significant in psoriasis. The findings of cohort research involving 1959 subjects revealed that serum γ-glutamyltranspeptidase (GGT) levels gradually increased by blood mercury quartiles [29]. And GGT is commonly elevated in patients with psoriasis.

As an observational study, confounding is probably to blame for the positive correlation between mercury exposure and psoriasis, especially for fish, which is the primary source of mercury exposure [30]. Interestingly, a recent pooled analysis suggested that compared to controls, people living with psoriasis frequently exhibit unbalanced dietary habits, such as a higher fat intake and a lower intake of fish or nutritional fibers [31]. These results suggested that cofounding fish cannot explain the connection between mercury exposure and psoriasis. Otherwise, psoriasis and cardiovascular diseases have shared risk factors, including reactive oxygen species formation. While mercury may cause reactive oxygen species to be produced, which are linked to the onset of psoriasis and cardiovascular problems, a marginally inverse relationship was also found between mercury exposure and cardiovascular illnesses in Health Professionals Follow-up Study (NHS/HPFS), and the risk of cardiovascular disorders was 0.85 (0.72–1.01) for comparing to the fifth quintile of mercury exposure with the first quintile [32]. Nonetheless, a recent meta-analysis revealed that the risk of numerous cardiovascular endpoints regularly increases at a hair mercury content of 2 μg/g [33]. These results suggested that in future experimental research, amounts of mercury exposure should be considered when assessing the mercury-mediated oxidative stress and activation of the cellular protective mechanism. Thus, more research on the possible pathways is warranted regarding the scant data.

The potential mechanisms underlying the positive association between mercury exposure and psoriasis are unknown. Mercury is a heavy metal that is highly poisonous and bioaccumulative. It is responsible for neurotoxic chemical buildup, lipid peroxidation, and mitochondrial damage to proteins and DNA [34]. Chronic exposure to mercury leads to an increase in oxidative stress [35, 36]. On the other hand, mercury could increase the expression of the antioxidant genes for glutamate-cysteine ligase, manganese-superoxide dismutase, copper, zinc-superoxide dismutase, thioredoxin reductase one mRNA, and catalase as a cellular defense mechanism against oxidative stress, and direct contact with the cysteine residues of Keap1 and Akt/ glycogen synthase kinase three beta/Fyn pathway to activate the antioxidant signaling system.

According to our findings, this positive association between blood mercury and psoriasis appears to be more pronounced in non-diabetic adults. Psoriasis is an autoimmune skin disease. The possibility of multiple variables causing conditions to develop simultaneously is not precluded. Psoriasis frequently coexists with cardiovascular disease, diabetes mellitus, and metabolic syndrome [37]. Other studies, on the other hand, refuted this conclusion. The findings of cohort research involving 17,272 Americans show that in logistic regression models, psoriasis was not linked to diabetes, CVD, stroke, or microvascular disorders [38]. According to earlier studies, men and women saw similar rates of psoriasis prevalence: 2.8% (95% CI, 2.4%-3.3%) in men and 3.2% (95% CI, 2.6%-3.8%) in women [39]. Thus, more epidemiological research based on racial stratification analysis is required to determine the causes.

This is the first investigation into the relationship between total blood mercury levels and psoriasis in American adults. Large participant numbers from 1 successive NHANES cycle are another strength of this study. Using a sophisticated multi-stage probability sampling design, which improves the study’s representativeness and dependability, is one of its most vital points. Additionally, there are a few restrictions. Because of the cross-sectional design, causality cannot be established. Second, since hair or toenail mercury is unavailable, total blood mercury has been adopted as a biomarker of mercury exposure. Thirdly, although the diagnosis of psoriasis comes from a medical professional or health care practitioner, reliance on self-reported psoriasis conditions can lead to reporting bias and lack of clinical validation. In addition to this, the small sample size of patients diagnosed with psoriasis may lead to biased results. Lastly, even though we considered many possible confounders, residual confounding is still potential because of the study design. Yet, there were similarities in the outcomes between Models 1 and 2.

## Conclusions

Our study found an independently positive association between blood total mercury and psoriasis in US adults. Our research may shed light on potential psoriasis prevention and treatment strategies. Additional high-quality prospective studies are required to support our findings on this research problem.

## Supporting information

S1 Data Detailed description of survey data used. (DOCX)

S2 Data Availability data for this study. (XLS)

S1 Table Checklist of items that should be included in reports of cross-sectional studies. (DOCX)

## Abbreviations

NHANES: National Health and Nutrition Examination Survey—
CVD: Cardiovascular disease
NCHS: National Center for Health Statistics
BMI: Body mass index
LDL-C: low-density lipoprotein cholesterol)
GPx: glutathione peroxidase
OR: odds ratio
CI: confidence interval
GGT: serum γ-glutamyltranspeptidase
PIR: Income-to-Poverty Ratio
NHS/HPFS: Health Professionals Follow-up Study
