# Supplementary material for: Association between blood total mercury and psoriasis: The NHANES 2005–2006 and 2013–2014: A cross-sectional study
Source: PLoS One. 2024 Oct 15;19(10):e0309147. doi: 10.1371/journal.pone.0309147 (PMC11478867; doi:10.1371/journal.pone.0309147)
Supplement: S1 Data — (DOCX) [file pone.0309147.s001.docx]

**Supporting Information**

The National Health and Nutrition Examination Survey (NHANES) is a program of studies designed to assess the health and nutritional status of adults and children in the United States. The survey is unique in that it combines interviews and physical examinations. NHANES is a major program of the National Center for Health Statistics (NCHS)[1]. NCHS is part of the Centers for Disease Control and Prevention (CDC) and has the responsibility for producing vital health statistics for the Nation. The NHANES interview includes demographic, socioeconomic, dietary, and health-related questions. The examination component consists of medical, dental, and physiological measurements, as well as laboratory tests administered by highly trained medical personnel [2].

Our analysis included metal exposures and psoriasis measured over three NHANES cycle years: 2005–2006 and 2013–2014. Whole blood mercury was analyzed via inductively coupled plasma-dynamic reaction mass spectrometry (ICP-DRC-MS) at the CDC’s National Center for Environmental Health[3]. Dilution of the blood in the sample preparation step prior to analysis is a simple dilution of 1 part sample + 1 part water + 48 parts diluent. The effects of the chemicals in the diluent are to release metals bound to red blood cells making them available for ionization, reduce ionization suppression by the biological matrix, prevent clogging of the sample introduction system pathways by undissolved biological solids, and allow introduction of internal standards to be utilized in the analysis step. Tetramethylammonium hydroxide (TMAH, 0.4% v/v) and Triton X-100TM (0.05%) in the sample diluent solubilizes blood components. Triton X-100TM also helps prevent biological deposits on internal surfaces of the instrument’s sample introduction system and reduce collection of air bubbles in sample transport tubing. Ammonium pyrrolidine dithiocarbamate (APDC) in the sample diluent (0.01%) aids in solubilizing metals released from the biological matrix. Ethyl alcohol in the sample diluent (1%) aids solubility of blood components and aids in aerosol generation by reduction of the surface tension of the solution. The internal standards, rhodium, iridium, and tellurium, are at a constant concentration in all blanks, calibrators, QC, and samples. Monitoring the instrument signal ratio of a metal to its internal standard allows correction for instrument noise and drift, and sample-to-sample matrix differences. Liquid samples are introduced into the mass spectrometer through the inductively coupled plasma (ICP) ionization source. The liquid diluted blood sample is forced through a nebulizer which converts the bulk liquid into small droplets in an argon aerosol. The smaller droplets from the aerosol are selectively passed through the spray chamber by a flowing argon stream into the ICP. By coupling radio-frequency power into flowing argon, plasma is created in which the predominant species are positive argon ions and electrons and has a temperature of 6000-8000 K. The small aerosol droplets pass through a region of the plasma and the thermal energy vaporizes the liquid droplets, atomizes the molecules of the sample and then ionizes the atoms. The ions, along with the argon, enter the mass spectrometer through an interface that separates the ICP (at atmospheric pressure, ~760 torr) from the mass spectrometer (operating at a pressure of 10-5 torr). The ions first pass through a focusing region, then the dynamic reaction cell (DRC), the quadrupole mass filter, and finally are selectively counted in rapid sequence at the detector allowing individual isotopes of an element to be determined.

In compliance with CDC recommendations, all statistical analyses used suitable NHANES sampling weights that took into consideration the intricate sampling design and several rounds of cluster surveys[4]. Weighted Student's t-tests or weighted chi-square tests were used to assess the differences between blood total mercury for continuous data and categorical variables, respectively. Because of the complex, multistage probability sampling design of NHANES, an inferential statistics method was used to represent the large nationally representative sample. Thus, we summarize categorical parameters as a proportion by logistic regression analyses. To examine the association between blood total mercury and psoriasis, weighted multivariable regression models were employed in three different models. In model 1, no covariates were adjusted. Model 2 was adjusted for sex, age, and race. Model 3 was adjusted for age, gender, race, serum glucose, serum total bilirubin, White blood cell count, total cholesterol, triglyceride, LDL-cholesterol, HDL-cholesterol, Body Mass Index, waist Circumference, cadmium, lead, education, annual family income, drinking alcohol, diabetes, high blood pressure, and smoking. Smooth curve fittings were employed to address the non-linearity. Additionally, subgroup analysis of the associations between blood total mercury and psoriasis was conducted using stratified multivariable logistic regression models with stratified factors including sex, age, BMI, and diabetes. Moreover, stratified factors were considered as possible effect modifiers. To assess the heterogeneity using the likelihood ratio test, an interaction term was used[5]. For the variables that show a normal distribution, we use mean interpolation, while for variables that show a skewed distribution, we use median interpolation. All analyses were performed utilizing R version 4.1.3 (http://www.R-project.org, Te R Foundation) and Empower software (www. empowerstats.com; X&Y Solutions, Inc., Boston MA). Statistical signifcance was considered to exist at a two-sided P<0.05.

**References**

[1] W. Zhang, S.F. Peng, L. Chen, H.M. Chen, X.E. Cheng, and Y.H. Tang, Association between the Oxidative Balance Score and Telomere Length from the National Health and Nutrition Examination Survey 1999-2002. Oxid Med Cell Longev 2022 (2022) 1345071.

[2] T.D. Cheng, C. Ferderber, B. Kinder, and Y.J. Wei, Trends in Dietary Vitamin A Intake Among US Adults by Race and Ethnicity, 2003-2018. Jama 329 (2023) 1026-1029.

[3] A.P. Sanders, M.J. Mazzella, A.J. Malin, G.M. Hair, S.A. Busgang, J.M. Saland, and P. Curtin, Combined exposure to lead, cadmium, mercury, and arsenic and kidney health in adolescents age 12-19 in NHANES 2009-2014. Environ Int 131 (2019) 104993.

[4] C.L. Johnson, R. Paulose-Ram, C.L. Ogden, M.D. Carroll, D. Kruszon-Moran, S.M. Dohrmann, and L.R. Curtin, National health and nutrition examination survey: analytic guidelines, 1999-2010. Vital Health Stat 2 (2013) 1-24.

[5] Z. Qin, D. Du, Y. Li, K. Chang, Q. Yang, Z. Zhang, R. Liao, and B. Su, The association between weight-adjusted-waist index and abdominal aortic calcification in adults aged ≥ 40 years: results from NHANES 2013-2014. Sci Rep 12 (2022) 20354.
